# Recurrence Rates of Intraosseous Ameloblastoma Cases With Conservative or Aggressive Treatment: A Systematic Review and Meta-Analysis

**DOI:** 10.3389/fonc.2021.647200

**Published:** 2021-05-19

**Authors:** Xue Qiao, Junxiu Shi, Jiayi Liu, Jinwen Liu, Yan Guo, Ming Zhong

**Affiliations:** ^1^ Department of Central laboratory, School and Hospital of Stomatology, China Medical University, Liaoning Province Key Laboratory of Oral Disease, Shenyang, China; ^2^ Department of Oral Biology, School and Hospital of Stomatology, China Medical University, Liaoning Province Key Laboratory of Oral Disease, Shenyang, China; ^3^ Department of Developmental Cell Biology, Cell Biology Division, Key Laboratory of Cell Biology, Ministry of Public Health, Key Laboratory of Medical Cell Biology, Ministry of Education, China Medical University, Shenyang, China; ^4^ Department of Oral Histopathology, School and Hospital of Stomatology, China Medical University, Liaoning Province Key Laboratory of Oral Disease, Shenyang, China; ^5^ Department of Periodontics, School and Hospital of Stomatology, China Medical University, Liaoning Province Key Laboratory of Oral Disease, Shenyang, China; ^6^ Department of Stomatology, Xiang’an Hospital of Xiamen University, Xiamen, China

**Keywords:** intraosseous ameloblastomas, conservative treatment, meta-analysis, aggressive treatment, recurrence rates

## Abstract

**Objective:**

This study aimed to systematically investigate and compare the post-treatment recurrence of intraosseous ameloblastoma in patients treated with conservative or aggressive approaches.

**Methods:**

Systemic searches of PubMed, Medline, Cochrane Library, and Embase databases from inception to October 28, 2020, were conducted. Studies that aimed to evaluate the recurrence of intraosseous ameloblastoma by conservative and aggressive treatment approaches were included.

**Results:**

A total of 20 studies with 942 ameloblastoma cases were included. Fourteen studies included patients with ameloblastoma who received conservative treatment, and 16 studies reported the overall recurrence rate for patients undergoing aggressive treatment. The pooled results indicated that the recurrence rate for aggressive treatment [0.12, 95% confidence interval (CI) = 0.09–0.16] was significantly lower than that for conservative treatment, with a recurrence rate of 0.30 (95% CI = 0.23–0.39). Similar results were obtained when stratifying the participants by the histological classification. When trying stratification analysis following the original included studies, multicystic ameloblastoma presented a much higher recurrence rate than solid and unicystic ameloblastomas.

**Conclusion:**

These findings supported the hypothesis that aggressive treatment might lead to a lower recurrence rate than conservative treatment. More studies and meta-analyses following the new histological classification of ameloblastomas are needed to validate and support the findings.

## Introduction

Ameloblastomas are benign but locally invasive neoplasms that represent 10% of all jaw tumors (1). They are characterized by slow growth, asymptomatic swelling and/or perforation of the cortical bone. However, without any treatment, ameloblastomas might grow into massive proportions, causing facial deformity (2).

Surgery is deemed to be one of the major treatment approaches for ameloblastomas, and resection is considered an ideal surgical method (3). However, resection involves a wide bone margin, leading to the immediate or delayed bony reconstruction of the defect with tissue grafts and/or prosthetic rehabilitation. Meanwhile, with aggressive treatment and the current standard of care, a high degree of morbidity is observed and the risk of recurrence still exists (4). Recurrent ameloblastoma is difficult to be treated, especially if it recurs in an anatomical region with limited surgical access or is detected in a later stage.

A systematic review and meta-analysis was conducted by Antonoglou on the recurrence rates of intraosseous ameloblastomas of the jaws (5) who compared the conservative *versus* aggressive treatment approaches. The researchers categorized ameloblastomas following the 2004 World Health Organization (WHO) classification version, which were also termed as “conventional” ameloblastomas according to the current classification, into solid/multicystic, unicystic, and peripheral; they strongly recommended resection as the preferred treatment choice for both unicystic and solid/multicystic ameloblastomas. Recently, several studies (6–9) were conducted on this topic and provided new evidence, wherein some studies (10–12) reported inconsistent results by comparing with previous meta-analyses (5). The newly published literature makes it possible to complete an updated, with more power of persuasion, and well-conducted systematic review and meta-analysis. Therefore, this systematic review and meta-analysis was re-conducted to investigate the post-treatment recurrence of intraosseous ameloblastoma in cases treated with conservative or aggressive approaches.

## Materials and Methods

### Literature Search

PubMed, Medline, Cochrane Library, and Embase were searched for potentially relevant studies without any language and time restriction from their inception till October 28, 2020. The following individual and joint keywords were used to search potential studies: “ameloblastoma” OR “adamantoblastoma” AND “recurre” OR “recurrence” OR “reverse” OR “reappear”. To include more relevant studies, the bibliographies of all relevant studies and reviews were also searched. Google Scholar was also searched for relevant studies. This meta-analysis was conducted according to the Preferred Reporting Items for Systematic Reviews and Meta-analysis guidelines (13).

### Eligibility Criteria

Studies that met the following inclusion criteria were included for data analysis: (1) study population diagnosed with ameloblastomas; (2) patients with ameloblastoma who received treatment and focused on ameloblastoma recurrence; (3) necessary data that could be extracted from original studies; (4) studies published in English; and (5) the study providing detailed information or a newly published study selected if the study population from the same institution was reported in duplicate.

Case reports, letters, reviews, comments, conference abstracts, and studies conducted in animal models or experiments *in vitro*, studies in languages other than English, and studies without available data were excluded from this meta-analysis.

### Data Extraction

The selection process was evaluated according to the inclusion criteria and independently conducted by two authors (XQ, JL). If insufficient information was available from abstracts, then the authors reviewed the full-texts of the studies. All necessary information from the standard-compliant studies was extracted using a standardized form by two reviewers independently, and a consensus was reached on all items by a discussion with a third reviewer (JS). The information, such as study characteristics (first author, year of publication, and study design), participant’s characteristics (mean age and male percentage), and disease characteristics, and follow-up period and recurrence rates, was extracted from each study.

### Quality Scoring of Studies

The overall quality of evidence of the included studies was assessed independently by two authors following the Quality Assessment and Validity Tool of Newcastle–Ottawa Scale (NOS), which is used for assessing the methodological quality of meta-analysis of observational studies (14).

The grades of NOS for observational studies were based on three factors: participant selection, comparability of study participants, and exposure of factors. The detailed criteria for the three factors were as follows: representation of cases, process of selection and definition for controls, comparability of cases and controls based on the design or analysis, ascertainment of exposure, same method of ascertainment for cases and controls, and nonresponse rate if the cases were defined adequately. A study was given a maximum of one star for each numbered item within the selection and exposure categories and a maximum of two stars for comparability. The score ranged from two to nine points. A scale of less than two points indicated poor quality, three to five points denoted medium quality, and six to nine indicated high quality. Sensitivity analysis was conducted if the studies were assessed as low or medium quality according to the NOS.

### Statistical Analysis

The recurrence rates with corresponding 95% confidence intervals (CIs) were calculated. Inverse variance methods with random-effects models were used to pool the results of the included studies. Stratification analysis on the histological classification of the cases was done following the original included studies. The standard heterogeneity test based on *I* ^2^ statistics was used to assess the consistency of the effect sizes. Heterogeneity was categorized as with and without significant heterogeneity according to the values of *I* ^2^ ≥50% and <50% (15), respectively. To explore the sources of heterogeneity, the enrolled studies were sequentially excluded to observe the overall impact of the individual study. The publication bias was assessed using Begg’s rank correlation (16) and Egger’s weighted regression methods (17). Statistical analyses and the Begg’s and Egger’s tests were performed using Stata 15.0 (Stata Co., TX, USA). A *P* value of <0.05 indicated a statistically significant difference.

## Results

### Study Selection

**Figure 1:**
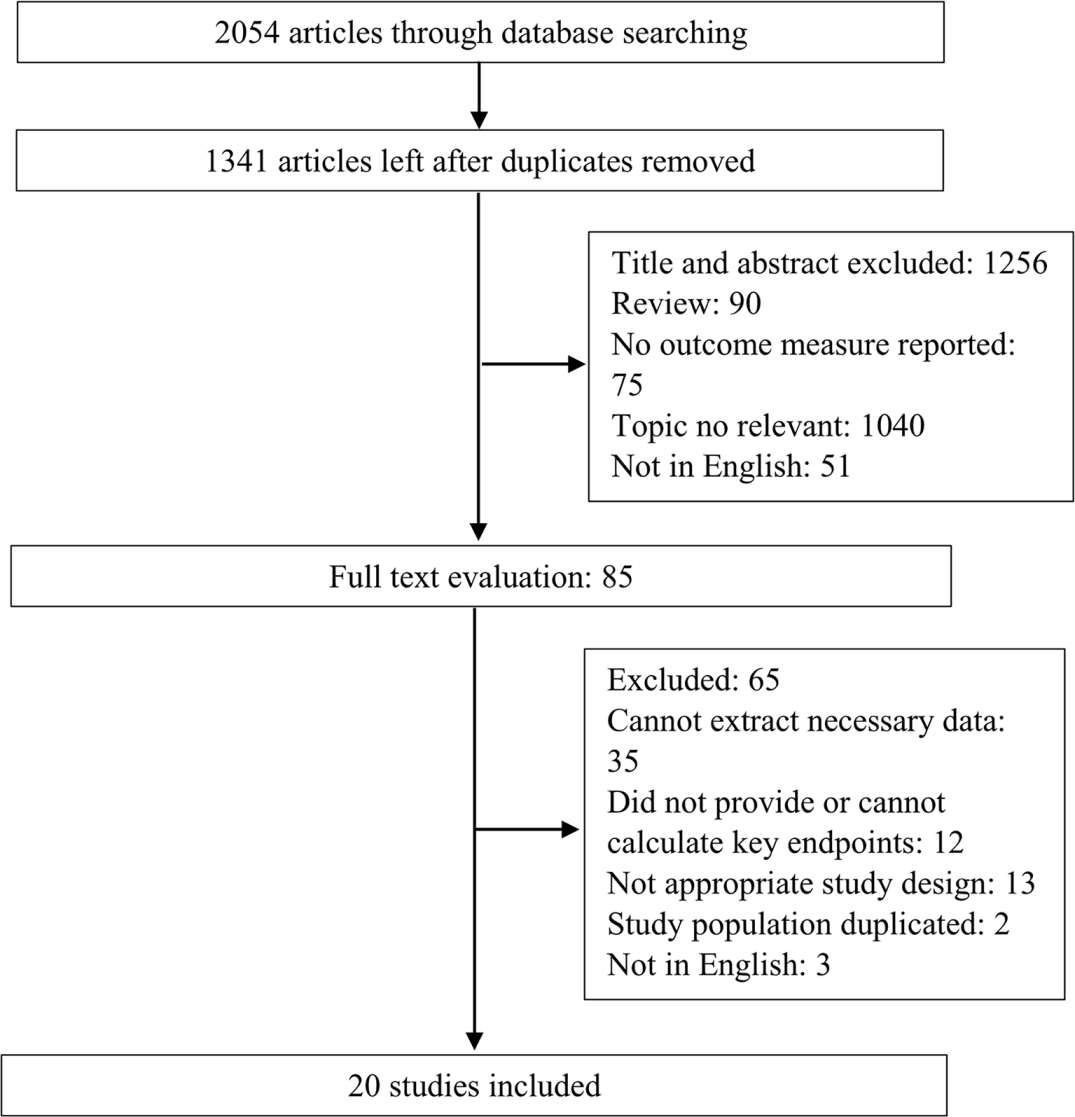
Flowchart of the study selection process.

The flowchart of the study selection process is presented in **Figure 1**. A systematic literature search yielded 2,054 studies, and 713 of these were excluded because they were duplicates. By strictly following the aforementioned inclusion and exclusion criteria, 1,256 abstracts and titles were reviewed initially. After studying the full-texts of 65 studies, 20 (6–12, 18–24) were finally included for data extraction and meta-analysis.

### Study Characteristics

Overall 20 studies with 942 ameloblastoma cases were included, which were published between 1977 and 2019. The sample size ranged from 10 to 234. The studies were conducted in the USA (18, 19), Brazil (20), Nigeria (21), Italy (9, 10, 22), Japan (23, 24), Jordan (25), Netherlands (8, 26), China (12, 27, 28), India (6), Sri Lanka (29), Singapore (11), Australia (30), and South Korea (28). Of the 20 included studies, 10 comprised patients who accepted conservative or aggressive treatments, four included patients who accepted only conservative treatment, and six included patients who accepted only aggressive treatment. The conservative treatment strategies used in the included studies comprised conservative surgery, enucleation, and cryosurgery. The aggressive treatment strategies included radical surgery, marginal resection, segmental resection, resection with bone margin, and enucleation plus peripheral ostectomy.

### Quality Assessment of Studies

According to the NOS scale, the quality of included studies was acceptable. Five studies were assessed as high quality (≥eight points), 15 studies were assessed as moderate quality (six to eight points), and none of the studies were assessed as low quality. The detailed scores for each included study are shown in **Supplementary Table 1**.

### Ameloblastoma Patients Who Received Conservative Treatment

**Table 1** presents the characteristics of the participants who received conservative treatment, and the cases were categorized according to the second or third version of the ameloblastoma histological classification published by the WHO. In a majority of the studies, the participants were followed up for more than 50 months.

**Table 1:** Study and participant characteristics with conservative treatment.

Studies included	Country	Follow-up time (means)	Included cases	Recurrence cases	Treatment form	Histological classification
Robinson et al. (18)	USA	106.2 months	20	3	Enucleation	Unicystic
Leider et al. (19)	USA	90 months	33	1	Enucleation or curettage	Unicystic
Curi et al. (20)	Brazil	90.5 months	2	0	Curettage/Cryosurgery	Unicystic
			29	8	Curettage/cryosurgery	Solid
Olaitan et al. (21)	Nigeria	99.1 months	11	2	Enucleation and primary closure	Unicystic
Nakamura et al. (22)	Japan	NA	14	2	Marsupialization alone/Marsupialization followed by enucleation and curettage	Unicystic
			22	10	Marsupialization followed by enucleation and curettage/Enucleation plus curettage	Solid
Chapelle et al. (26)	Netherlands	111.6 months	4	0	Enucleation/Enucleation with application of Carnoy’s solution	Unicystic
Lee et al. (27)	China	74 months	24	4	Enucleation/Enucleation/Carnoy’s Solution	Unicystic
Hong et al. (28)	Korea	96 months	104	40	Conservative	Multicystic
			67	11	Conservative	Unicystic
Migaldi et al. (10)	Italy	57 months	1	0	Conservative surgery	Unicystic
Krishnapillai et al. (6)	India	10−192 months^a^	27	2	Enucleation/Curettage	Unicystic
Darshani et al. (29)	Sri Lanka	NA	56	20	Enucleation	Multicystic
			43	12	Enucleation	Unicystic
Hertog et al. (8)	Netherlands	96 months	8	7	Enucleation	Solid
			8	4	Enucleation	Multicystic
			6	3	Enucleation	Mixed
			6	3	Enucleation	Unicystic
Hasegawa et al. (24)	Japan	8−130 months^a^	23	10	Enucleation after Marsupialization/Enucleation/Curettage/Enucleation/Curettage	Multicystic
Zheng et al. (12)	China	3−72 months^a^	16	11	Enucleation	Unicystic

NA, not available.

a Range of follow-up time.

**Figure 2:**
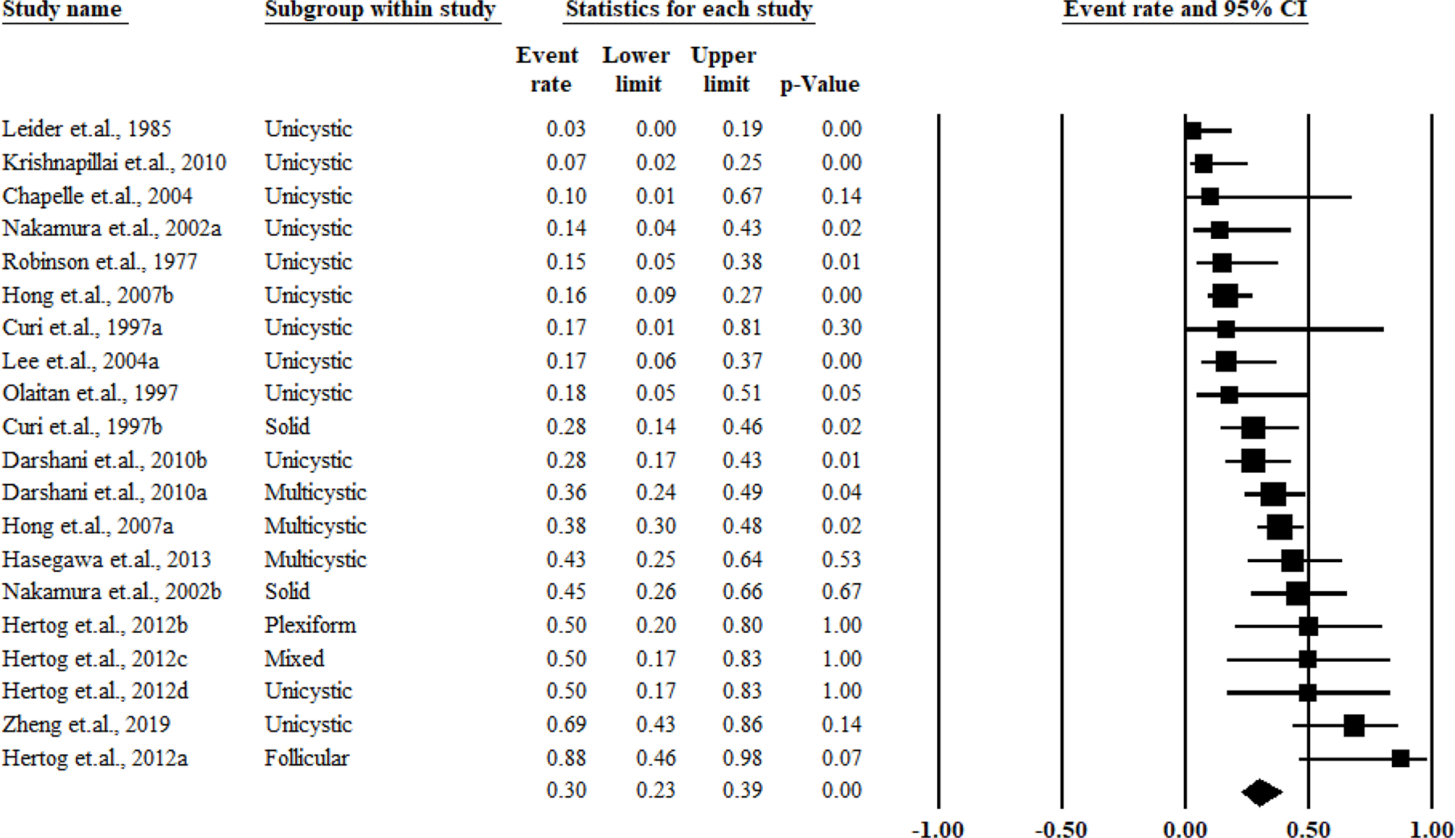
Summary of the conservative treatment recurrence rate.

**Figure 3:**
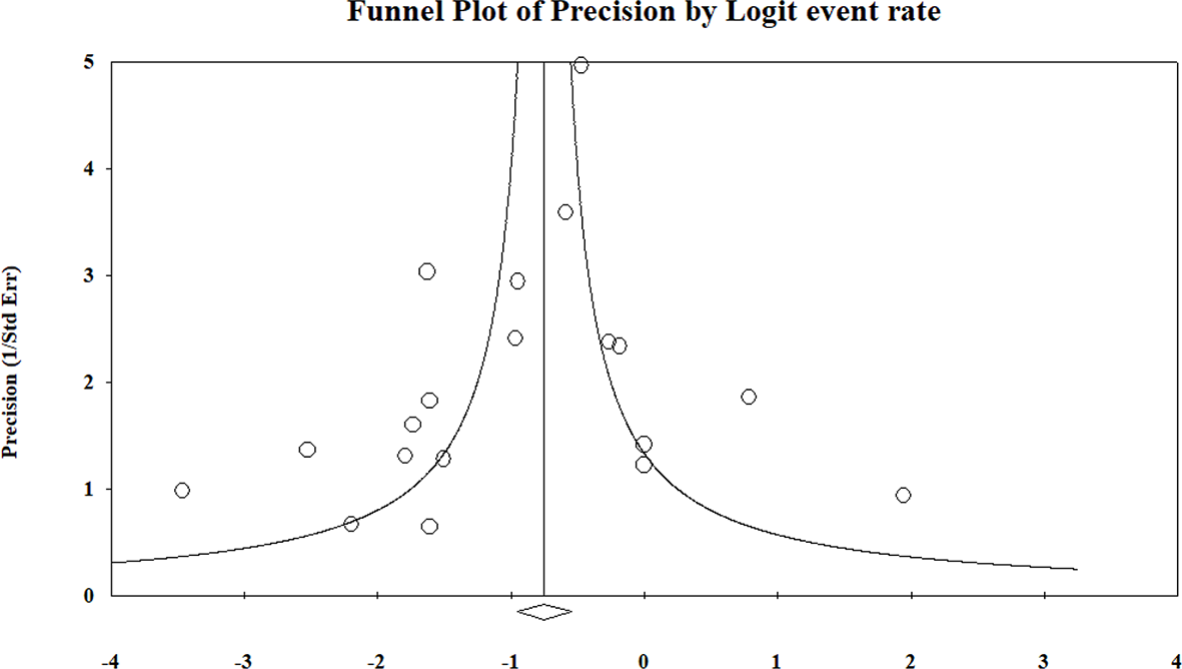
Summary of the funnel plot of conservative treatment recurrence rate.

**Figure 4:**
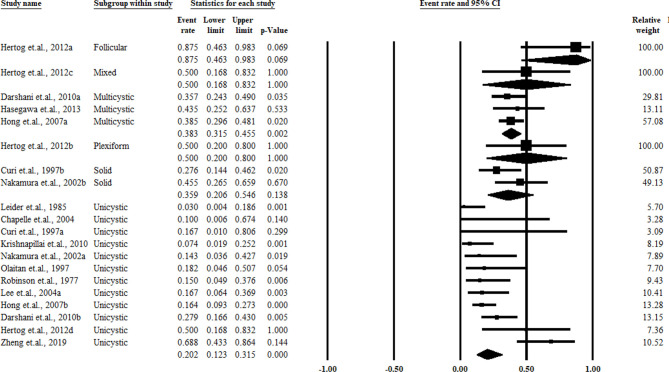
Summary of the conservative treatment recurrence rate stratified by the histological classification.

The pooled results for the recurrence rate were as high as 0.30 (95% CI = 0.23–0.39) and showed no significant heterogeneity (*I* ^2^ = 0%). When stratifying the participants based on the histological classification, multicystic tumors showed a higher recurrence rate of 0.38 (95% CI= 0.32–0.46, *I* ^2^ = 21%) compared with that of solid (0.36, 95% CI = 0. 21–0.55, *I* ^2^ = 32%) and unicystic tumors (0.20, 95% CI = 0.12–0.32, *I* ^2^ = 0%). The pooled results and forest plots for all conservative treatment cases are shown in **Figure 2**, and the funnel plot is shown in **Figure 3**. More detailed stratified results by the histological classification are presented in **Figure 4**.

### Ameloblastoma Patients Receiving Aggressive Treatment

The included studies that reported the overall recurrence rates for aggressive treatment cases are shown in **Table 2**. Similar to conservative treatment cases, the cases were categorized following the second or third version of ameloblastoma the histological classification published by the WHO. In a majority of the studies, the participants were followed up for more than 50 months.

**Table 2:** Study and participant characteristics with aggressive treatment.

Studies included	Country	Follow-up time (means)	Included cases	Recurrence cases	Treatment form	Histological classification
Curi et al. (20)	Brazil	90.5 months	5	2	Resection/Cryosurgery	Solid
Olaitan et al. (21)	Nigeria	99.1 months	10	1	Resection of a lesion with the encompassing dentoalveolar process and preservation of the lower border of the mandible	Unicystic
Becelli et al. (23)	Italy	NA	42	0	Marginal resection (radical)/Segmental resection	Solid
			18	0	Marginal resection	Unicystic
Nakamura et al. (22)	Japan	NA	13	0	Radical surgery	Unicystic
			29	3	Radical surgery	Solid
Al-Khateeb et al. (25)	Jordan	91.2 months	6	0	Enucleation plus peripheral ostectomy and resection	Unicystic
			2	0	Enucleation plus peripheral ostectomy and resection	Solid
			2	0	Enucleation plus peripheral ostectomy and resection	Mixed
Lee et al. (27)	China	74 months	5	0	Resection with bone margin	Mural invasion
Hong et al. (28)	Korea	96 months	50	6	Resection with bone margin (radical)/Segmental resection	Multicystic
			13	0	Resection with bone margin/Segmental resection	Unicystic
Migaldi et al. (10)	Italy	57 months	12	3	Radical surgery	Multicystic
Zhang et al. (7)	China	3–60 months^a^	6	0	Segmental resection	Multicystic
			2	0	Segmental resection	Unicystic
Krishnapillai et al. (6)	India	10–192 months^a^	46	7	Wide margin resection	Multicystic
Darshani et al. (29)	Sri Lanka	NA	27	2	marginal, segmental, and total resection	Multicystic
			21	0	marginal, segmental, and total resection	Unicystic
Hertog et al. (8)	Netherlands	96 months	2	0	Radical surgery	Unicystic
			3	0	Radical surgery	Solid
			1	0	Radical surgery	Mixed
			1	0	Radical surgery	Unicystic
Bianchi et.al. (9)	Italy	53.6 months	27	0	Segmental resection	Multicystic
			4	0	Segmental resection	Unicystic
Ooi et.al. (11)	Singapore	59 months	24	0	Segmental resection	Multicystic
			6	0	Segmental resection	Unicystic
Singh et al. (30)	Australia	51 months	29	1	Radical surgery	Multicystic
			2	0	Radical surgery	Unicystic
Zheng et al. (12)	China	3–72 months^a^	10	4	Segmental resection	Unicystic

NA, not available.

a Range of follow-up time.

**Figure 5:**
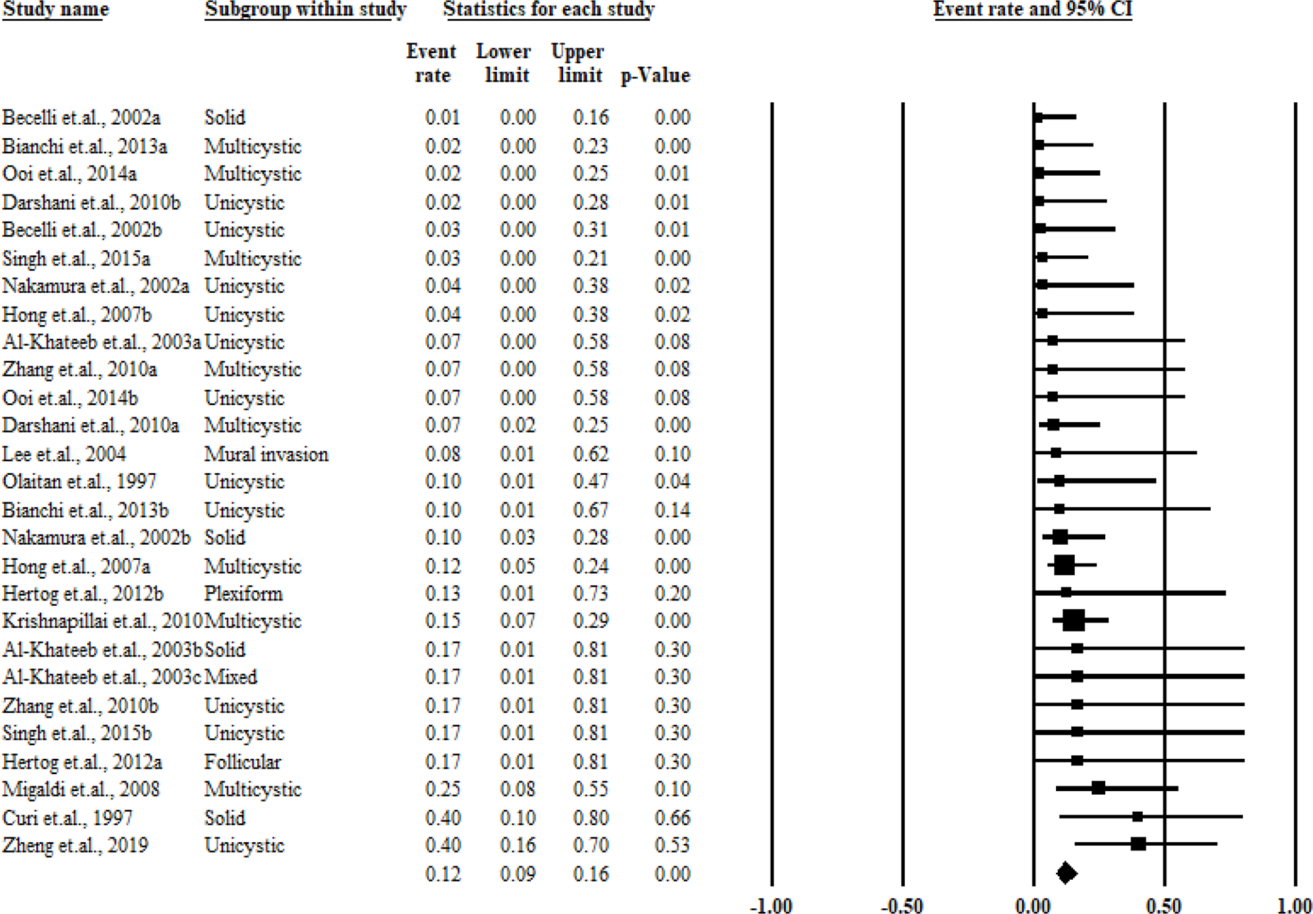
Summary of the aggressive treatment recurrence rate.

**Figure 6:**
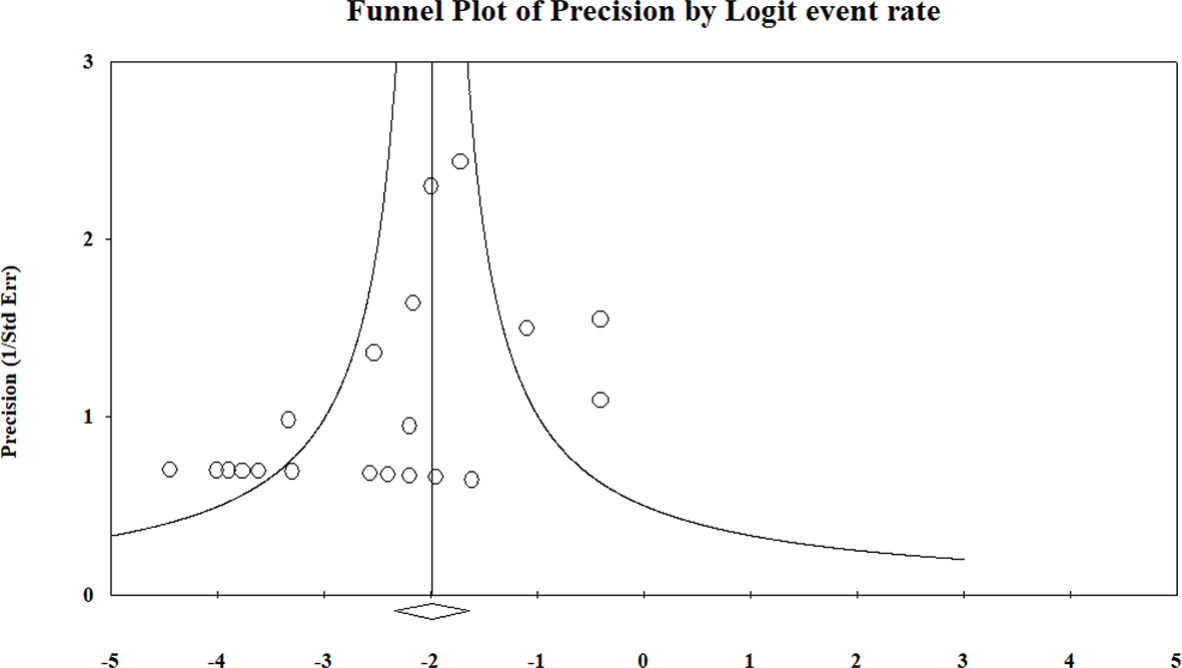
Summary of the funnel plot of aggressive treatment recurrence rate.

**Figure 7:**
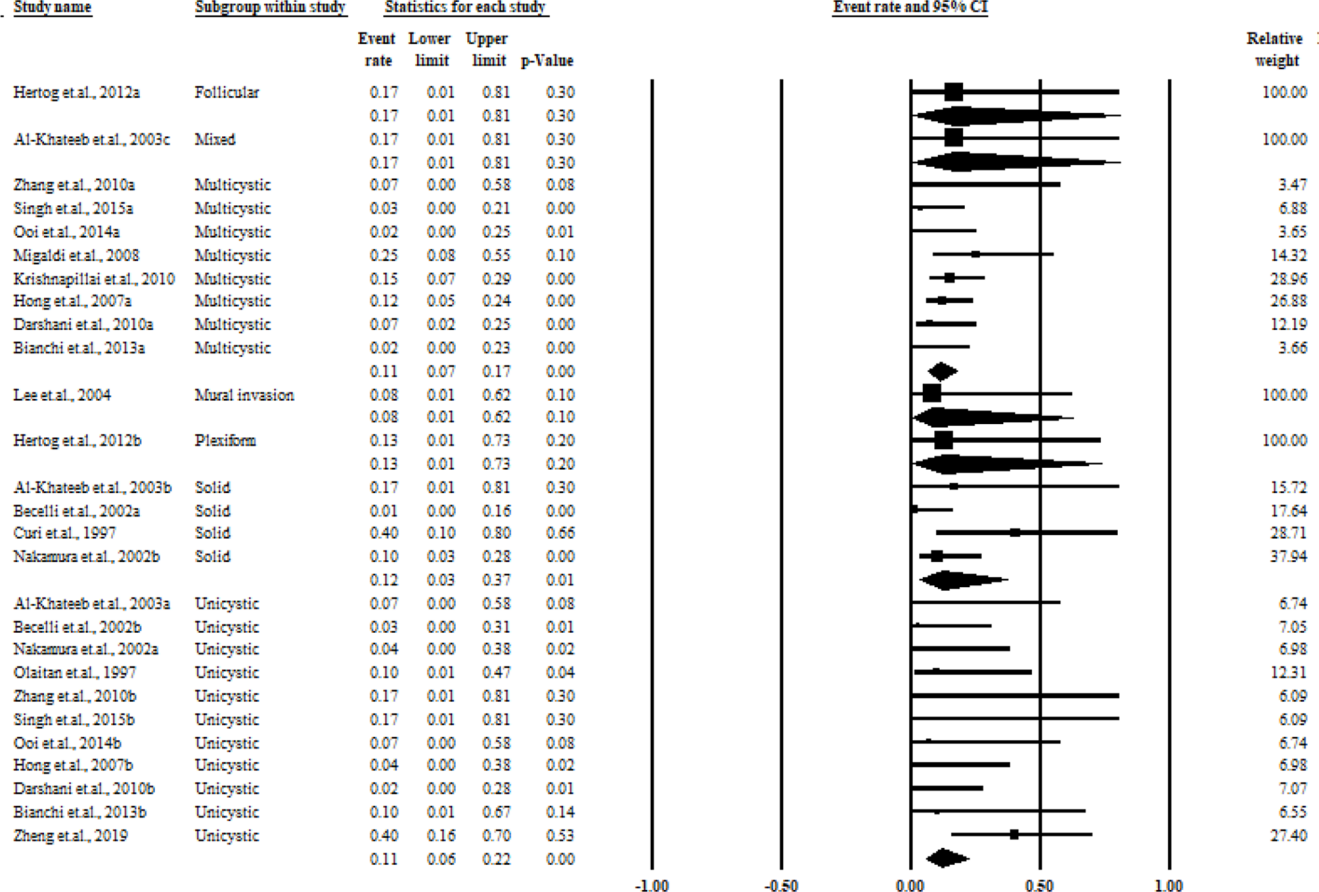
Summary of the aggressive treatment recurrence rate stratified by the histological classification.

As shown in **Figure 5**, the overall pooled recurrence rate for aggressive treatment was much lower than that for conservative treatment in cases with a recurrence rate of 0.12 [95% CI = 0.09–0.16), without any significant heterogeneity (*I* ^2^ = 0%)]. When stratifying the participants by the histological classification according to the original reports of the authors, aggressive treatment cases also showed lower recurrence rates as those of multicystic (0.11, 95% CI = 0. 07–0.17, *I* ^2^ = 0%), solid (0.12, 95% CI = 0.03–0.37, *I* ^2^ = 43%), and unicystic ameloblastomas (0.11, 95% CI = 0.06–0.22, *I* ^2^ = 21%). The funnel plot is presented in **Figure 6**, and more detailed stratified results based on the histological classification are presented in **Figure 7**.

### Publication Bias

No potential publication bias was detected among the included trials according to Begg’s rank correlation analysis and Egger’s weighted regression analysis (all pooled *P* values of >0.05). The detailed results of publication bias for each pooled process are shown in **Supplementary Table 2**.

## Discussion

A total of 20 studies comprising 942 patients with ameloblastoma were included for data extraction and meta-analysis. All studies had acceptable quality. Fourteen studies included patients with ameloblastoma who received conservative treatment, and 16 studies reported the overall recurrence rate for the aggressive treatment cases. The pooled results indicated that the recurrence rate for aggressive treatment (0.12, 95% CI = 0.09–0.16) was much lower than that for conservative treatment, with a recurrence rate of 0.30 (95% CI = 0.23–0.39). Similar results were observed when stratifying the participants by the histological classification. Multicystic ameloblastomas presented a much higher recurrence rate than solid and unicystic ameloblastomas.

In a previous meta-analysis conducted by Antonoglou (5), the summary of recurrence rates of unicystic and solid/multicystic ameloblastomas ranged from 0.2 to 12% and 0.8 to 38%, respectively. Compared with the previous meta-analyses, the number of included studies increased by one time. Both this study and the previous studies focused on the recurrence rates with regard to conservative and aggressive treatment in patients with ameloblastoma. In this study, similar results were obtained and the conclusion was better because solid ameloblastomas were best treated by resection rather than by conservative treatment. Another meta-analysis conducted by Hendra et al. (31) aimed to investigate the outcomes of radical and conservative treatment approaches, which partly reported the recurrence rates. However, the authors failed to summarize and assess the recurrence rates by stratification analysis according to the newest classification, that is, the 2017 WHO pathological category. In this meta-analysis, the authors reported the recurrence rates of 0.08 and 0.41 for patients using aggressive and conservative treatments, respectively. The significant differences in the pooled results might be due to the diverse study objectives and standard categories. A majority of conventional ameloblastomas were treated with segmental resection, including 1- to 2-cm bone margins and more than one adjacent uninvolved anatomic barrier for proper margins (32). Ameloblastomas treated with curettage by community dentists or resected by surgeons before a detailed histological workup might have been examined (32). In this setting, under-treatment and persistent biological behavior of ameloblastomas might lead to high recurrence rates and morbidity (33).

The results of this study showed that the recurrence rates was much lower for patients who received aggressive treatment than for patients who received conservative treatment. This finding can guide the management of treatment approaches and offer prognostic information (34). Moreover, in this study, multicystic ameloblastomas presented a significantly higher recurrence rate compared with solid and unicystic ameloblastomas. Hong et al. (28) conducted a study on age- and location-matched participants and reported that patients with multicystic ameloblastomas had a 3.02-fold greater chance of recurrence compared with patients with solid or unicystic ameloblastomas. In clinical settings, surgeons deemed that ameloblastoma treatment should initially be conservative and performed with radical surgery when necessary after a recurrence. Therefore, multicystic ameloblastoma cases may be treated with the complete removal of the tumor while preserving the lower portion of the mandible whenever possible. Future studies should focus on the long-term follow-up of postoperative multicystic ameloblastoma cases with panoramic radiographs regularly. At the same time, studies with methodological standardization are needed to explore and compare the treatment performance among various types of multicystic ameloblastomas. However, since the first edition of the WHO classification published in 1971, the version has been updated three times. In 2017, the new version simplified the classification into three types: conventional, unicystic, and peripheral. As the solid/multicystic type could be confused with the unicystic type, the term put forwarded in 2004 version was discarded. In this critical situation, more studies aiming to provide targeted therapies are warranted. Meanwhile, studies are needed to validate the recurrence rate with new classifications.

Compared with previous meta-analyses, the strengths of this study were that it included a larger number of studies and provided more persuasive recurrence rates on various types of multicystic ameloblastomas. Although all the included studies were of moderate or high quality, the limitations of the present meta-analysis could not be ignored while interpreting the results. First, the number of included studies was small, and all the studies were conducted in Western countries and focused on the Caucasian population. The results might have been affected by environmental, medical, and genetic factors, which only partially annotated the associations; also, the representativeness with regard to the target population might have been weakened. Second, the limited number of studies reported various treatment methods, impeding future investigations. Third, a majority of the studies were categorized according to the second or third version of the WHO histological classification. With the new version, the solid/multicystic term was discarded due to its confusion with the unicystic type. The current results might have led to misclassification. Fourth, potential language bias might exist because the literature search included only studies published in English. Fifth, publication bias could not be assessed for all analyses due to the small sample size.

In conclusion, in this meta-analysis, the recurrence rates of ameloblastoma in patients who received aggressive treatment and conservative treatment were first assessed. The pooled results indicated that the recurrence rate for aggressive treatment was much lower than that for conservative treatment. Similar results were also observed when stratifying the participants by the histological classification. Multicystic ameloblastoma demonstrated a much higher recurrence rate than solid and unicystic ameloblastomas. However, in the previously published data, none of the studies categorized the patients according to the 2017 WHO histological classification. Therefore, more studies and meta-analyses following the new histological classification of ameloblastomas are warranted to validate and support the findings.

## Data Availability Statement

The original contributions presented in the study are included in the article/**Supplementary Material**. Further inquiries can be directed to the corresponding authors.

## Author Contributions

MZ conceived and designed the study. XQ and JiaL conducted most of the experiments. XQ and JS wrote the manuscript. YG and MZ reviewed and approved the manuscript. All authors contributed to the article and approved the submitted version.

## Funding

This study was supported by the National Natural Science Foundation of China (81072197, 81470758, and 81972535), the Natural Science Foundation of Liaoning Province (2019-ZD-0787), and the Youth Project Foundation of the Department of Education of Liaoning Province (QN2019021).

## Conflict of Interest

The authors declare that the research was conducted in the absence of any commercial or financial relationships that could be construed as a potential conflict of interest.
