# Supplementary material for: Recurrence Rates of Intraosseous Ameloblastoma Cases With Conservative or Aggressive Treatment: A Systematic Review and Meta-Analysis
Source: Front Oncol. 2021 May 19;11:647200. doi: 10.3389/fonc.2021.647200 (PMC8170394; doi:10.3389/fonc.2021.647200)
Supplement: Supplementary file 1 [file Table_1.docx]

**Supplementary Table 1. Quality assessment of included studies by Newcastle-Ottawa Scale^a^**

| **Studies included** | **Overall quality score** |
| --- | --- |
| Robinson et.al., 1977 | 6 |
| Leider et.al., 1985 | 6 |
| Curi et.al., 1997 | 6 |
| Olaitan et.al., 1997 | 7 |
| Becelli et.al., 2002 | 6 |
| Nakamura et.al., 2002 | 6 |
| Al-Khateeb et.al., 2003 | 7 |
| Chapelle et.al., 2004 | 6 |
| Lee et.al., 2004 | 6 |
| Hong et.al., 2007 | 8 |
| Migaldi et.al., 2008 | 8 |
| Krishnapillai et.al., 2010 | 7 |
| Darshani et.al., 2010 | 7 |
| Zhang et.al., 2010 | 7 |
| Hertog et.al., 2012 | 7 |
| Hasegawa et.al., 2013 | 8 |
| Bianchi et.al., 2013 | 7 |
| Ooi et.al., 2014 | 8 |
| Singh et.al., 2015 | 7 |
| Zheng et.al., 2019 | 8 |

^a^, the study quality was assessed according to the Newcastle Ottawa Quality assessment scale for cross-sectional studies or cohort studies. This scale assigns a maximum of 9 points to each study.
