# Supplementary material for: Recurrence Rates of Intraosseous Ameloblastoma Cases With Conservative or Aggressive Treatment: A Systematic Review and Meta-Analysis
Source: Front Oncol. 2021 May 19;11:647200. doi: 10.3389/fonc.2021.647200 (PMC8170394; doi:10.3389/fonc.2021.647200)
Supplement: Supplementary file 2 [file Table_2.docx]

**Supplementary Table 2. Publication bias of summarized outcomes**

| **Outcomes** | **Begg (*P* value)** | **Egger (*P* value)** |
| --- | --- | --- |
| Summarized conservative treatment recurrence rate (overall) | 0.61 | 0.71 |
| Summarized aggressive treatment recurrence rate (overall) | 0.60 | 0.35 |
| Summarized conservative treatment recurrence rate (multicystic) | 0.56 | 0.32 |
| Summarized conservative treatment recurrence rate (solid) | 0.33 | 0.41 |
| Summarized conservative treatment recurrence rate (unicystic) | 0.21 | 0.30 |
| Summarized aggressive treatment recurrence rate (multicystic) | 0.71 | 0.76 |
| Summarized aggressive treatment recurrence rate (solid) | 0.61 | 0.59 |
| Summarized aggressive treatment recurrence rate (unicystic) | 0.17 | 0.21 |
